# Face pareidolia is enhanced by 40 Hz transcranial alternating current stimulation (tACS) of the face perception network

**DOI:** 10.1038/s41598-023-29124-8

**Published:** 2023-02-04

**Authors:** Annalisa Palmisano, Giulio Chiarantoni, Francesco Bossi, Alessio Conti, Vitiana D’Elia, Serena Tagliente, Michael A. Nitsche, Davide Rivolta

**Affiliations:** 1 Department of Education, Psychology, and Communication, University of Bari Aldo Moro, Bari, Italy; 2 IMT School for Advanced Studies Lucca, Lucca, Italy; 3 Department of Psychology and Neurosciences, Leibniz Research Center for Working Environment and Human Factors (IfADo), Dortmund, Germany; 4 Department of Neurology, University Medical Hospital Bergmannsheil, Bochum, Germany; 5 School of Psychology, University of East London (UEL), London, UK

**Keywords:** Human behaviour, Perception, Neurophysiology

## Abstract

Pareidolia refers to the perception of ambiguous sensory patterns as carrying a specific meaning. In its most common form, pareidolia involves human-like facial features, where random objects or patterns are illusionary recognized as faces. The current study investigated the neurophysiological correlates of face pareidolia via transcranial alternating current stimulation (tACS). tACS was delivered at gamma (40 Hz) frequency over critical nodes of the “face perception” network (i.e., right lateral occipito-temporal and left prefrontal cortex) of 75 healthy participants while completing four face perception tasks (‘Mooney test’ for faces, ‘Toast test’, ‘Noise pareidolia test’, ‘Pareidolia task’) and an object perception task (‘Mooney test’ for objects). In this single-blind, sham-controlled between-subjects study, participants received 35 min of either *Sham*, *Online*, (40Hz-tACS_ON), or *Offline* (40Hz-tACS_PRE) stimulation. Results showed that face pareidolia was causally enhanced by 40Hz-tACS_PRE in the Mooney test for faces in which, as compared to sham, participants more often misperceived scrambled stimuli as faces. In addition, as compared to sham, participants receiving 40Hz-tACS_PRE showed similar reaction times (RTs) when perceiving illusory faces and correctly recognizing noise stimuli in the Toast test, thus not exhibiting hesitancy in identifying faces where there were none. Also, 40Hz-tACS_ON induced slower rejections of face pareidolia responses in the Noise pareidolia test. The current study indicates that 40 Hz tACS can enhance pareidolic illusions in healthy individuals and, thus, that high frequency (i.e., gamma band) oscillations are critical in forming coherent and meaningful visual perception.

## Introduction

Pareidolia refers to the illusory perception of meaningful shapes from random or ambiguous stimuli. In the visual domain, individuals often report seeing images of faces, animals, or objects in random scenes, such as the Man in the Moon, the Moon Rabbit, and Jesus in a toast^1^. From an evolutionary perspective, among the multitude of random stimuli we perceive in everyday life, others’ faces represent the most relevant stimuli for social relations, and our visual system seems to be particularly sensitive to facial configurations^2–4^. Thus, individuals’ tendency to see human faces in clouds, mountains, or rocky discontinuities (i.e., face pareidolia) might be linked to our innate preference for face-like stimuli^5^, as well as to the existence of a face-detection system across primates^6,7^. It should be noted that monkeys do not experience pareidolia as humans do, which could be due to a human-unique aptitude for anthropomorphizing objects with face-like patterns^8^. As in real face perception, individual differences characterize face pareidolia^9,10^, with some people heavily perceiving face illusions while others reporting weaker effects^11–14^.

Spatio-temporal features of the human face perception system have been extensively investigated with neuroimaging techniques such as functional Magnetic Resonance Imaging (fMRI) and electro/magneto-encephalography (EEG/MEG)^15–17^. Face perception relies on a network of brain regions forming a “core” system (i.e., face-specific occipito-temporal areas)^18,19^, and an “extended” system of prefrontal regions^20–22^. Perception of *real* and *illusory* (i.e., pareidolic) faces show similar activity patterns^23^, with “core” regions being both quickly (within 200 ms) engaged by pareidolic faces (i.e., illusory faces seen from random noise) and real faces^1,24–28^. As seen in real face perception^29^, and face mental imagery^30^, face pareidolia requires the interaction between bottom-up and top-down paths (i.e., between visual areas and prefrontal regions)^1^, to resolve perceptual decisions under uncertainty^31^, and give rise to conscious perception^32^. Furthermore, high-frequency (> 30 Hz) and low-amplitude neural oscillations in the gamma-band range (GBO)^33^, as recorded with EEG/MEG^34–36^, represent a potential neurophysiological correlate of face pareidolia. In fact, GBO mediate the general construction of coherent perceptual representations based on the integration of visual information^37–39^.

Albeit the neuroimaging evidence reviewed above provides information about the localization and timing of face pareidolia, there is so far a lack of *causal* evidence of its neurophysiological underpinnings. Thus, the main aim of the current study is to test whether the exogenous entrainment of GBO within “core” and “extended” face regions via transcranial alternating current stimulation (tACS), by facilitating perceptual grouping and visual integration, can causally enhance pareidolic face illusions in a sample of healthy volunteers. This would be in line with neurophysiological evidence indicating that clinical populations experiencing frequent face-pareidolia, such as first-episode psychotic patients, are characterized by cortical hyperexcitability^40^ and enhanced GBO^41^ in face-sensitive regions. tACS consists in delivering a weak sinusoidal electric current between two or more scalp electrodes^42^ that can be applied at biologically relevant frequencies. In line with the view that tACS selectively interacts with endogenous brain oscillations and related functions^43,44^, frequency-tuned tACS can affect, for instance, visual perception^45,46^, memory^47–49^, problem-solving^50,51^ and high-level visual cognition^52^.

The study of illusory face perception is rendered complex by the literature inconsistencies on the concept of face pareidolia and its operationalization. Indeed, it has been used to refer to faces seen in face-like patterns in the environment, as well as in meaningless noise patterns. Accordingly, previous studies investigated pareidolia with pure noise images (i.e., in which no face is present)^1^, images with patterns resembling faces^53^, pictures of environments with and without face-like patterns^54^, and Mooney stimuli (i.e., black and white images from photographs of faces taken in a dark-contrasted environment vs. scrambled)^12,55^. These latter have been consistently adopted in the literature, as the perception of Mooney stimuli is at the interplay between sensory processing, mental imagery, and visual working memory^56,57^. In light of this heterogeneity, we adopted different tasks potentially catching pareidolia from multiple viewpoints. Both the frequency rate of illusory (i.e., non-existing) perceived faces^53^, as well as changes in reaction times (RTs)^58^, indicate pareidolia occurrence.

In line with previously reported tACS effects on both accuracy and RTs (e.g., visual detection tasks)^59,60^, we hypothesized that tACS in the gamma (i.e., 40 Hz) frequency over critical face nodes of the “core” (e.g., electrode PO8 over the right occipito-temporal face-sensitive areas) and “extended” (e.g., electrode FP1 over the prefrontal cortex (PFC)) face network causally enhances face pareidolia in healthy participants. In other words, we expected 40 Hz tACS to cause more and/or faster “face answers” from scrambled/random-noise visual stimuli. Furthermore, since previous research has never directly investigated the effects of *timing* of tACS on visual cognition, we tested the behavioural effects of both *online* (i.e., tACS during task execution) and *offline* (i.e., tACS before task execution) neuromodulation (see^61^ for a similar approach).

## Methods

### Participants

A sample of 75 healthy volunteers (37 females; mean age 22.36 ± 2.42 SD) with normal or corrected-to-normal vision, and without any recorded history of psychiatric or neurological disorders were recruited for this single-blind, sham-controlled, between-subjects study. All participants were naïve to the research hypotheses and the experimental conditions. Participants were assigned to one of three groups receiving different stimulation protocols (see the section below). Prior to the testing session, they received a verbal and written explanation of the procedure and the potential adverse effects of brain stimulation (e.g., itching and tingling skin sensation, skin reddening, headache). Participants gave their written informed consent to participate. The study was conducted according to the ethical standards of the World Medical Association Declaration of Helsinki, and the study protocol received approval from the Ethics Committee of the University of Bari ‘Aldo Moro’ (protocol number: ET-19-01).

### Experimental design

Participants were divided into three groups, receiving *sham*, or *online* (40Hz-tACS_ON) and *offline* (40Hz-tACS_PRE) 40 Hz tACS. Counterbalancing was applied within the Mooney tests, Noise pareidolia test, and Pareidolia task, with Mooney tests administered consecutively. Participants were assigned to one of three gender-matched groups of 25 participants each, receiving sham, 40Hz-tACS_ON, and 40Hz-tACS_PRE, respectively. All participants completed five tasks requiring circa 30 min of completion (see below for details on counterbalancing). Since females show similar cortical excitability as males only during the follicular phase of the menstrual cycle (when progesterone levels are low and estrogen levels are high), they were tested during this phase^62^. Tasks were set up with SuperLab 5.0 (Version 5.0.5, Cedrus Corporation, USA) and administered on a Fujitsu computer running Windows 10, with a 1920 × 1080 pixels 23-inches monitor. Participants were seated at approximately 60 cm eye distance from the screen.

**Figure 1:**
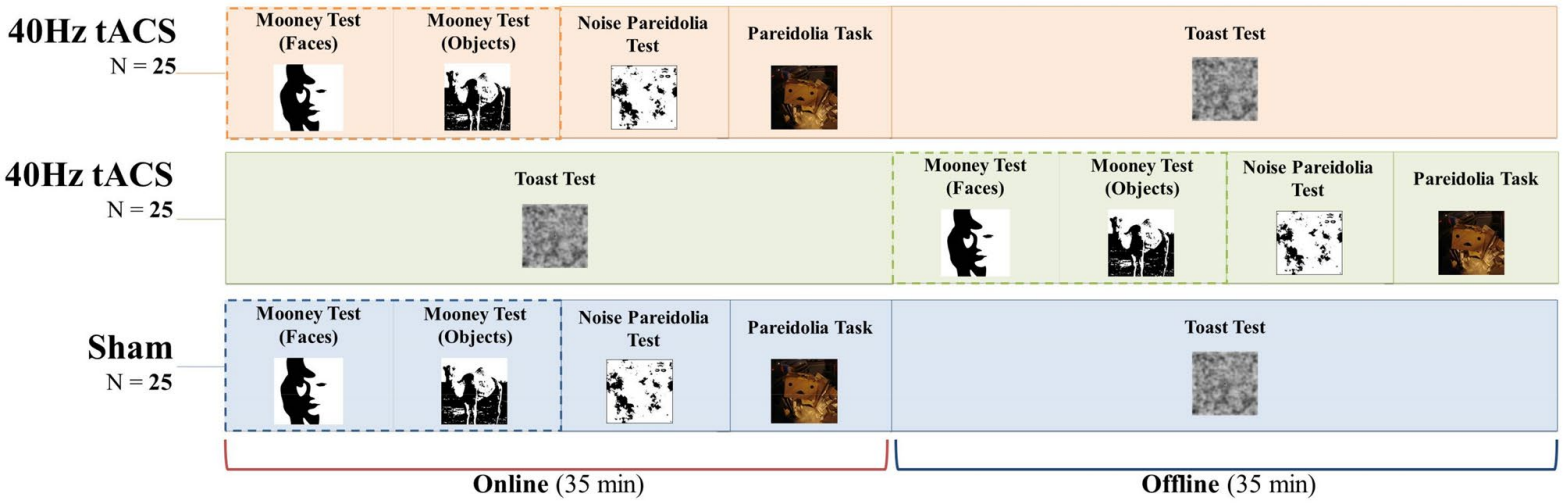
Schematic representation of the study design. Participants were divided into three groups, receiving sham, *online* (40Hz-tACS_ON) or *offline* (40Hz-tACS_PRE) 40 Hz tACS. Counterbalancing was applied within the Mooney tests, Noise pareidolia test, and Pareidolia task, with Mooney tests administered consecutively and counterbalanced between each other.

After signing informed consent, participants were invited to complete five tasks: (i) the Mooney test for faces^63^, (ii) the ‘Toast test’^1^, (iii) the Noise pareidolia test^53^, and (iv) the ‘Pareidolia task’. The (v) Mooney test for objects was also administered to monitor for potential category-specific effects (see below for tasks description and results). In order to align the experimental design with tasks’ length (i.e., the Toast test lasting circa 30 min, which represents the time taken to complete the remaining four tasks), grouping was designed as follows: 25 participants performed the Toast test during tACS (i.e., *online*), while Mooney tests (faces and objects), Noise pareidolia test, and Pareidolia task were completed *offline* (in a counterbalanced order, with the Mooney tests administered consecutively); whereas 25 participants completed the blocks in the opposite order, that is, *online* Mooney tests (faces and objects), Noise pareidolia test, and Pareidolia task (in a counterbalanced order, with Mooney tests administered consecutively), and *offline* Toast test. Participants receiving sham stimulation (N = 25) randomly performed either the Toast test *online* or *offline,* following the structure of the real tACS groups (Fig. 1). In each task, participants were asked to press the ‘M’ or ‘Z’ key on a computer keyboard to respond, respectively, whether they saw or not a face. The meaning of the response buttons was counterbalanced across subjects. At the end of the experimental session, all participants were debriefed and asked to fill in a ‘*tACS adverse effects questionnaire*’ about potential uncomfortable sensations experienced during or after the stimulation^64^.

### Mooney test for faces

**Figure 2:**
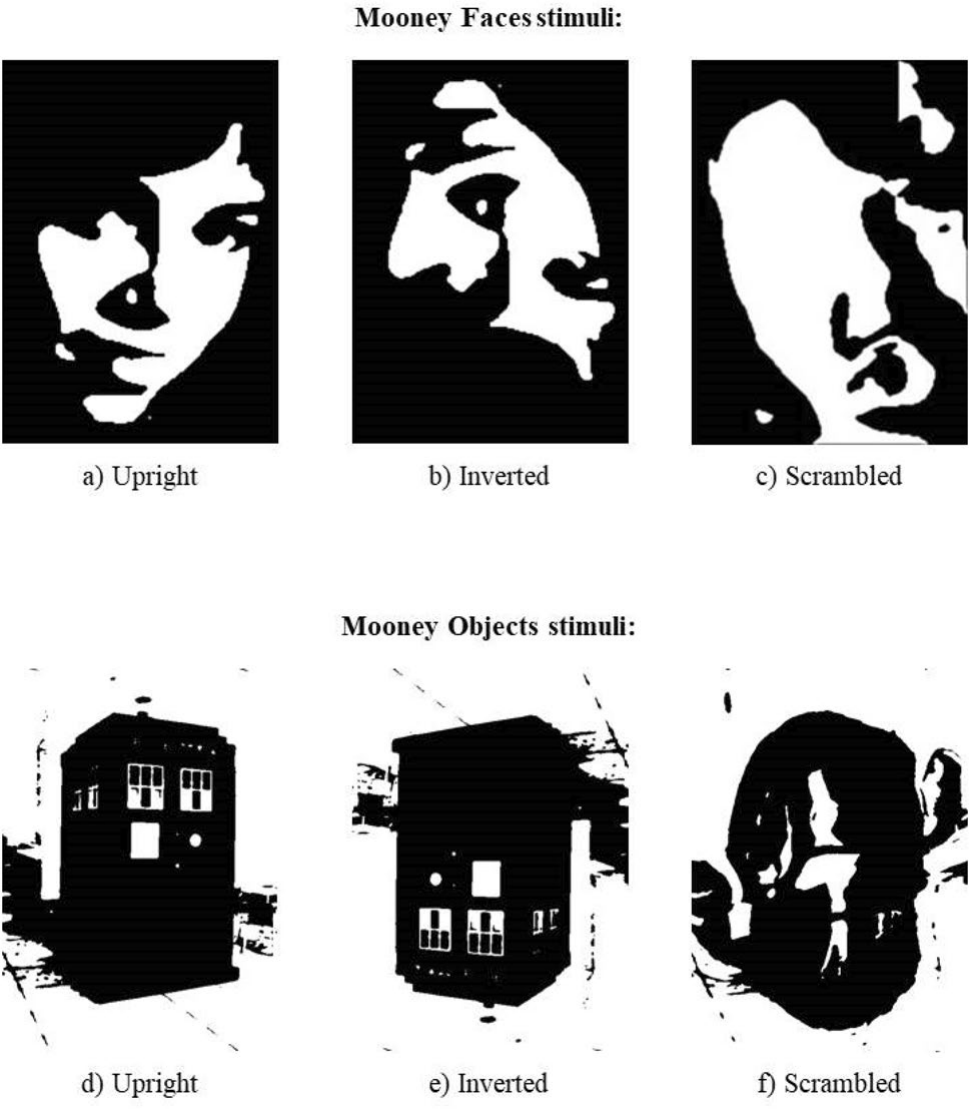
Sample stimuli in the Mooney test for faces (top), and the Mooney test for objects (bottom). Upright face (**a**), inverted face (**b**), and scrambled face (**c**); upright object (**d**), inverted object (**e**), and scrambled object (**f**)^63^.

The Mooney test for faces was created using black and white images from photographs of faces taken in a dark-contrasted environment^55^ (Fig. 2). A total of 180 stimuli (size: 160 × 230 pixels) were selected from the Schwiedrzik Database^63^. Participants were presented with three types of face stimuli: 45 upright (i.e., in canonic orientation), 45 inverted (i.e., upside-down), and 90 scrambled (meaningless images created from the face pictures). Randomized stimuli appeared for 350 ms, preceded by a 200 ms red fixation cross. Participants were asked to indicate, as fast as possible, if each stimulus contained a face or not. Correct answers were recorded if participants made their decision within 1200 ms from stimulus onset. A brief practice session with 10 trials was completed before the test. The tasks each took approximately 8 min to complete.

A higher rate of erroneous face detections versus correct scrambled recognitions (i.e., lower accuracy for scrambled stimuli), and/or longer RTs for no-face responses than face detections in participants receiving real stimulation than sham could reflect a tACS-driven pareidolia proneness in the Mooney test for faces.

### Mooney test for objects

The Mooney test for objects was created *ad-hoc* using photo-warp.com on pictures of various object categories (e.g., buildings, furniture, etc.), and following a standard procedure (i.e., increase of chromatic saturation, decrease of sharpness to remove details, and maximization of stimulus contrast)^40,63^, with a total of 180 stimuli (size: 160 × 230 pixels). The test structure was analogous to the Mooney test for faces (See Fig. 2 for sample stimuli). Since the Mooney test for objects was included as a control task to test the potential face-specificity of our tACS protocol, we expected no group differences in the rate of erroneous object detections between *sham* and *verum* stimulation.

### Toast test

**Figure 3:**
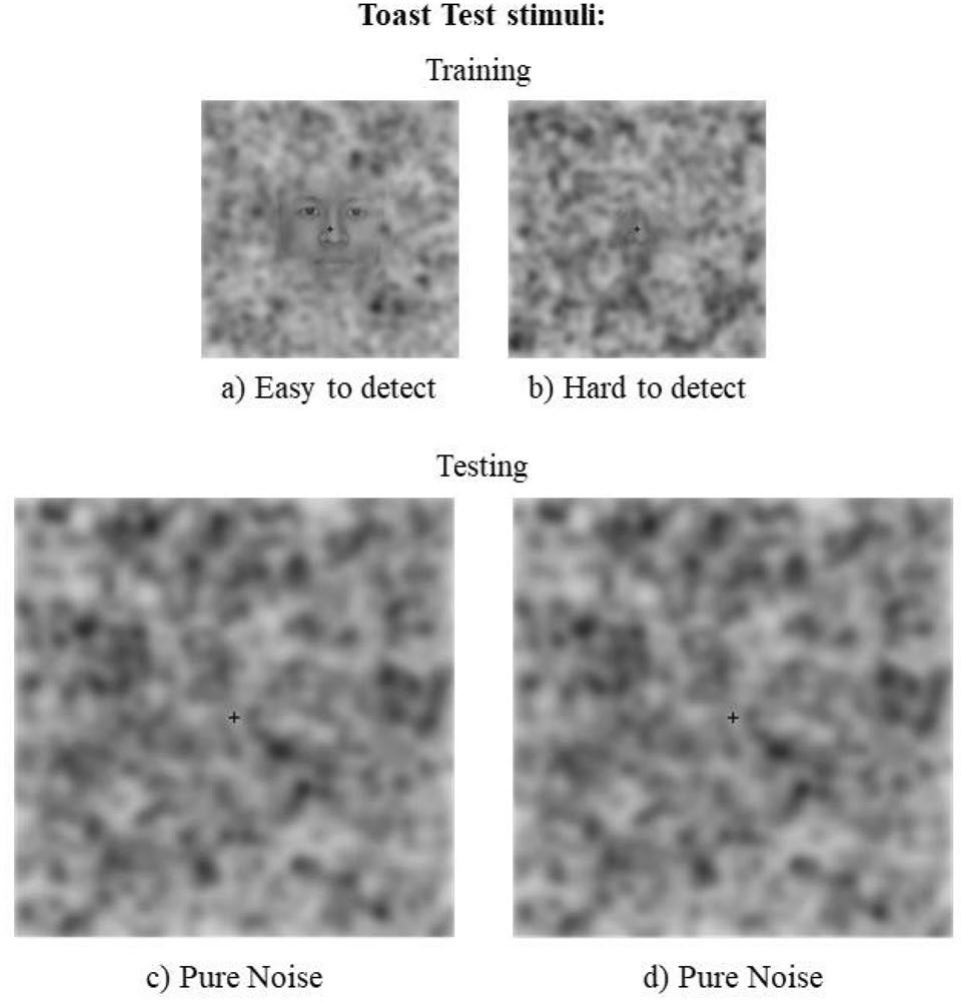
Sample stimuli in the Toast test. ‘Easy to detect’ (**a**) and ‘hard to detect’ (**b**) sample stimuli from the training phase; ‘pure noise’ (**c**, **d**) sample stimuli from the testing phase^1^.

The face pareidolia section of the Toast test was taken from Liu and colleagues^1^. This task includes training and a testing phase. The training was divided into three blocks of increasing difficulty; the first block included 20 easy-to-detect faces (well-defined hairless faces in the middle of visual noise) and 20 pure-noise images (white–gray spots on a black background); the second block comprised 20 hard-to-detect faces (little defined hairless faces in the middle of visual noise) and 20 pure-noise pictures, the third block contained 40 pure-noise images. Stimuli in each block were randomized for each participant. After the training phase, participants completed four testing blocks (120 stimuli per block). Although all stimuli in the testing session were pure-noise, participants were led to believe that 50% of them contained faces, with increasing levels of detection difficulty between sessions (Fig. 3). Both training and test stimuli were presented for 600 ms at the centre of the monitor, preceded by a 480 × 480 pixel checkerboard for 200 ms. At the beginning of each block, participants were instructed to decide, as fast as possible, if the stimulus contained a face or not. It took approximately 30 min to complete the task.

A higher rate of pareidolic responses versus correct noise detections, and/or shorted RTs for (always erroneous) pareidolic responses than noise detections in participants receiving real stimulation versus sham could reflect a tACS-driven pareidolia proneness in the Toast test.

### Noise pareidolia test

**Figure 4:**
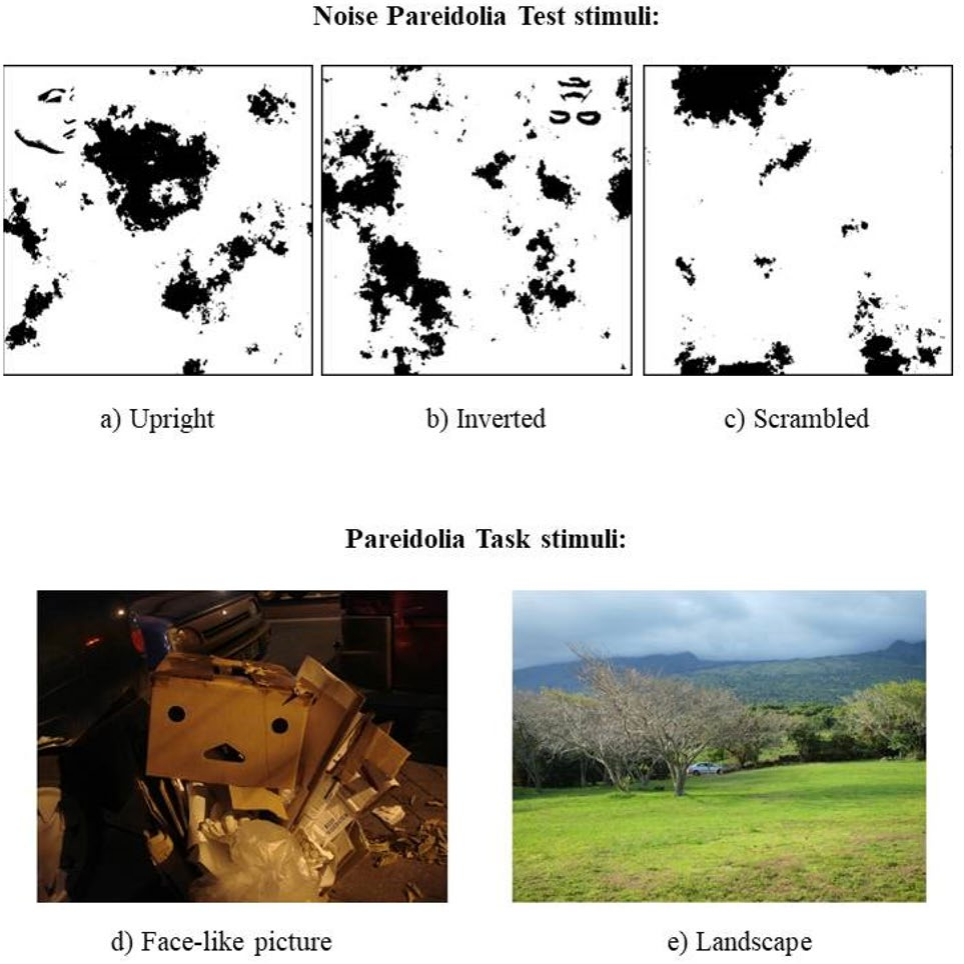
Sample stimuli from the Noise pareidolia test and Pareidolia task. Upper part of the figure: upright (**a**), inverted (**b**), and scrambled (**c**) stimuli from the Noise Parediolia test^53^. Bottom part of the figure: sample Face-like picture (**d**) and landscape (**e**) from the Pareidolia task.

An adapted version of the Noise pareidolia test^53^ was adopted. The stimuli were panels containing black and white spots (Fig. 4). In 20% of the cases, the spots were located across stylized, decentralized faces. A total of 80 stimuli were used: 64 pure noise, 8 upright faces, and 8 inverted faces. Randomized stimuli appeared for 200 ms and were preceded by a checkerboard presented for 200 ms. Participants indicated if the stimulus contained a face or not. Participants had 1200 ms to answer. A brief practice session was completed before the test. The task took approximately 5 min to complete.

A higher rate of erroneous face detections versus correct noise recognitions (i.e., lower accuracy for noise stimuli), and/or longer RTs for no-face responses than face detections in participants receiving real stimulation than sham could reflect a tACS-driven pareidolia proneness in the Noise pareidolia test.

### Pareidolia task

The Pareidolia task was composed of 100 coloured photos (50 face-like scenes and 50 scenes without any evident face-like pattern) (Fig. 4). A pilot study was conducted to validate the task. Eight volunteers were asked to decide if a face-like pattern was present or not in each picture (with no time limit). Since none of the images were classified in the wrong category, all stimuli were included in the task. Randomized stimuli appeared for 300 ms each. Participants indicated if they saw or not a face-like stimulus. They had to answer within 2000 ms after the end of the stimulus presentation when the screen was white. A brief practice session was completed before the actual test. The task took approximately 6 min to complete.

A higher rate of faces perceived in no-face images and/or shorter RTs for face- than no-face responses in participants receiving real stimulation than sham could reflect a tACS-driven pareidolia proneness in the Pareidolia task.

### tACS

40 Hz tACS was delivered by a battery driven, constant current stimulator (BrainSTIM stimulator; E.M.S. s.r.l.) via a pair of surface sponge electrodes (25 cm^2^) soaked in saline solution (0.9% NaCl) and applied to the scalp at the target location. Electrodes delivered an alternating current of 2 mA (peak to peak; current density: 0.08 mA/cm^2^) for 35 min. We adopted a bilateral bipolar-non balanced montage^65^. The sites of stimulation were identified using the International 10–20 EEG system, with one of the electrodes placed over PO8 (right occipito-temporal cortex–“core” face regions) and the other over FP1 (left PFC–“extended” face regions)^22,66^. In the Sham group, the stimulator was turned on for only 20 s. to elicit a short-lasting skin sensation. Overall, tACS applications in this study complied with safety guidelines^67^, and none of the participants reported major complaints or intolerable discomfort during or after tACS.

### Statistical analyses

All analyses were conducted using R Studio (R Team, 2015). Mixed-effects generalized linear models (GLMMs) on a binomial distribution were adopted to test for tACS effects on performance, with a critical α-error = 0.05. Descriptive statistics, results for random effects and fixed effects from each task are reported in the Supplementary Materials.

The experimental group (“Group”, 3 levels: sham, 40Hz-tACS_ON, 40Hz-tACS_PRE) served as between-subject independent variable. Participants’ “*Response”* (face-present responses vs. noise detections) was the dependent variable of the Toast test. Responses’ *Accuracy* served as a dependent variable for the Mooney tests for faces and objects, Noise pareidolia test, and Pareidolia task. The type of stimuli was included as an independent variable in interaction with Group for the Mooney tests (“*Stimuli*”: upright, inverted, scrambled), Noise pareidolia test (upright, inverted, noise), and Pareidolia task (faces, landscapes). The assessment of an increase/reduction of pareidolic illusions was derived from the Group*Stimuli interaction, revealing how participants accurately and rapidly perceived the stimuli as faces or not. The Mooney tests for faces and objects were analyzed separately as the two sets of stimuli (i.e., faces and objects) are not taken from the same database, with the latter being not yet validated.

Participants’ RTs were also evaluated as a dependent variable by using mixed-effects linear models (LMMs), with the same independent variables. All models used random intercepts on participants. Trials with RTs beyond ± 2 SD from the participant’s mean were discarded as potential outliers. The significance of each effect was estimated using the Satterthwaite approximation for degrees of freedom in LMMs and performing likelihood ratio tests (LRTs) with corresponding null models in GLMMs. Post-hoc comparisons (using Tukey HSD *p* value correction) were performed to probe statistically significant interactions.

### Uncomfortable sensation questionnaire

At the end of the experimental session, all participants were debriefed and asked to fill in a ‘tACS adverse effects questionnaire’ about potential uncomfortable sensations experienced during or after the stimulation protocol^64^. The *Uncomfortable Sensation Questionnaire* comprised 8 items, and participants were asked to assess their sensations through a scale ranging from 0 (‘lack of sensations’) to 4 (‘strong sensations’). Student’s *t*-tests performed on scores from the ‘tACS adverse effects questionnaire’ indicated a significant difference between groups, with participants in the tACS groups reporting, overall, higher discomfort sensations than the sham group *t*(73) = − 4.821, *p* < 0.001.

## Results

### Mooney tests for faces

**Figure 5:**
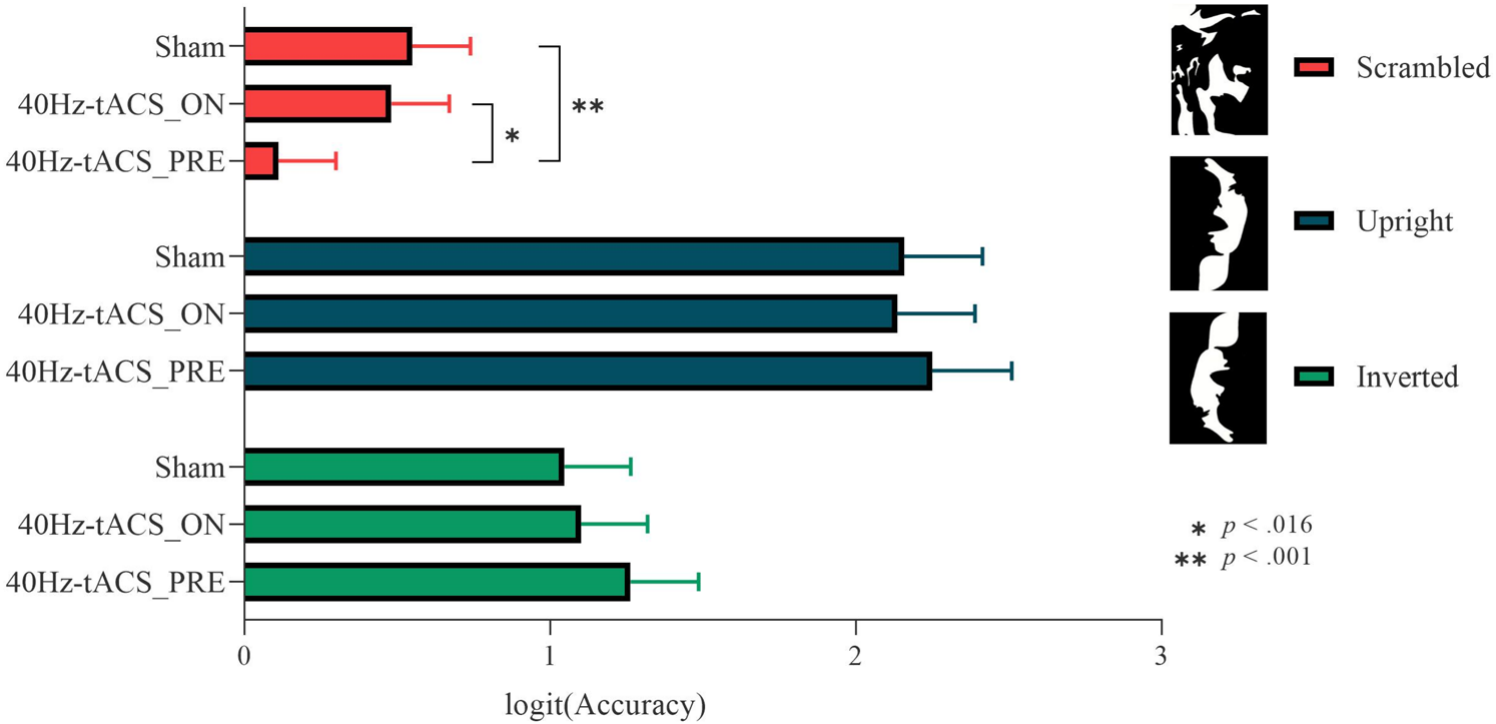
Group*Response interaction for the Mooney test for faces. Post-hoc contrasts based on Group. Participants in the 40Hz-tACS_PRE group exhibited worse performance in terms of accuracy for scrambled stimuli, which were more often misperceived as faces than in the Sham and 40Hz-tACS_ON groups (i.e., induced offline pareidolia proneness).

*Accuracy* data from the Mooney test (faces) showed a non-significant main effect of Group on Response (χ^2^(2) = 3.12, *p* = 0.210). The Group*Stimuli interaction on Response reached statistical significance (χ^2^(4) = 41.98, *p* < 0.001), with *post-hoc* contrasts based on Group showing that, for scrambled stimuli only, participants in the 40Hz-tACS_PRE group made more mistakes (mean = 0.526) than those in the sham (mean = 0.629) (*z* = 3.268, *p* < 0.001), and 40Hz-tACS_ON (mean = 0.615) (*z* = − 2.761, *p* = 0.016) groups, thus indicating that scrambled stimuli were more often erroneously perceived as real faces (Fig. 5). This demonstrates stronger face pareidolia (~ 10% increase of illusory face perceptions) after *offline* 40 Hz tACS. *Post-hoc* contrasts based on Stimuli showed significant differences in performance among all groups (all |*z|*s > 8; all *p*s < 0.001), with upright stimuli showing higher accuracy (sham = 0.890; 40Hz-tACS_ON = 0.890; 40Hz-tACS_PRE = 0.896; mean = 0.892) than inverted (sham = 0.734; 40Hz-tACS_ON = 0.745; 40Hz-tACS_PRE = 0.768; mean = 0.749), than scrambled stimuli (sham = 0.629; 40Hz-tACS_ON = 0.615; 40Hz-tACS_PRE = 0.526; mean = 0.59) (i.e., the well-known ‘Face Inversion Effect’ (FIE), an indirect index of holistic face processing^68,69^).

*RT* data showed a main effect of Stimuli (F(2,8983.9) = 815.849, *p* < 0.001). The Group*Stimuli interaction was also statistically significant (F(4,8983.9) = 21.112, *p* < 0.001). *Post-hoc* tests for the factor Stimuli showed differences in performance in all groups, in line with the FIE, with upright stimuli resulting in faster RTs than the inverted and scrambled ones (all |*z|*s > 4.5; all *p*s < 0.001). *Post-hoc* contrasts based on the factor Group did not reveal any differences (all |*z|*s < 1.5; all *p*s > 0.299).

### Mooney test for objects

**Figure 6:**
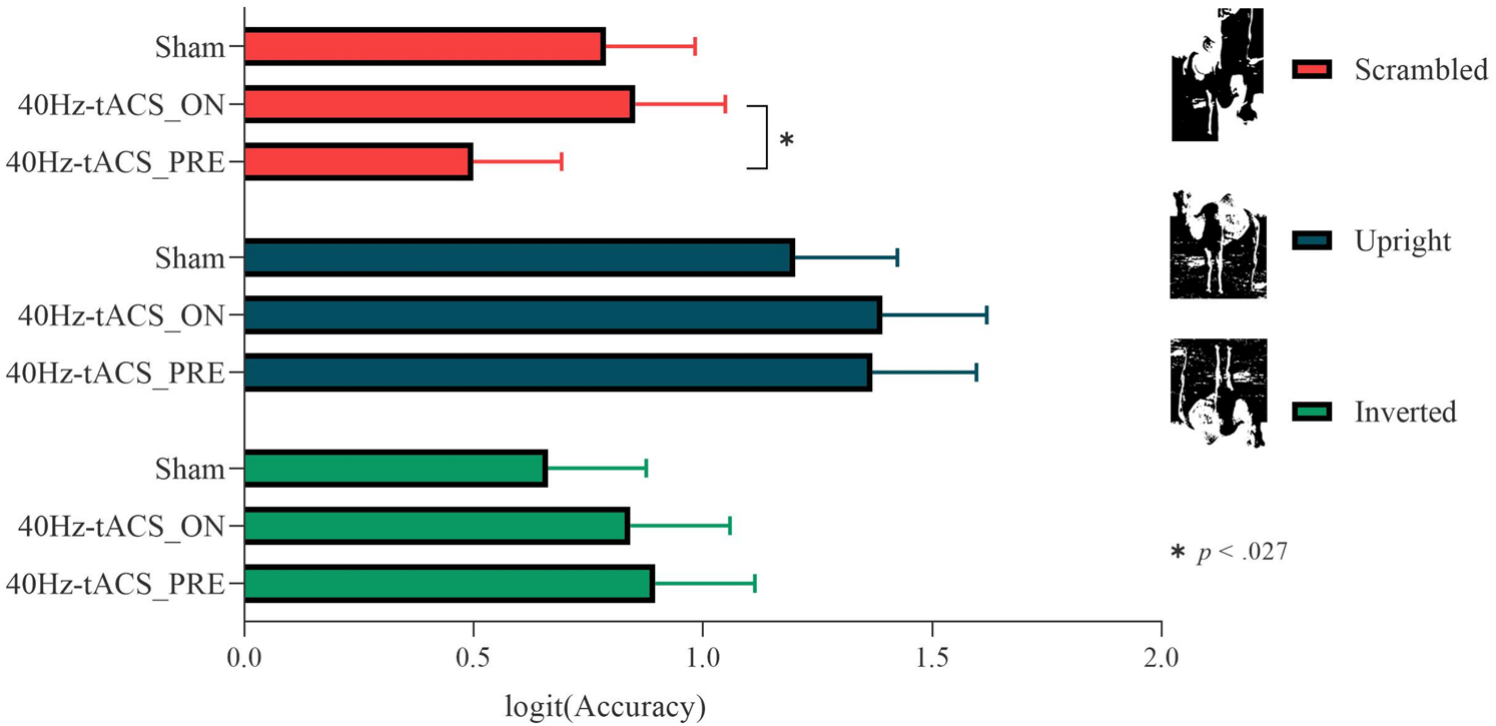
Group*Response interaction for the Mooney test for objects. Post-hoc contrasts based on Group. As compared to the Mooney test fo faces, no pareidolia proneness emerged by comparing performances from the 40Hz-tACS_PRE and the Sham groups. However, participants in the 40Hz-tACS_PRE group were significantly less accurate at recognizing scrambled stimuli than those in 40Hz-tACS_ON group.

*Accuracy* results showed a non-significant main effect of Group on Response (χ^2^(2) = 2, *p* = 0.366). The Group*Stimuli interaction effect on Response reached statistical significance (χ^2^(4) = 30.176, *p* < 0.001). P*ost-hoc* contrasts based on Group showed no difference between sham (mean = 0.680) and 40Hz-tACS_PRE (mean = 0.618) for scrambled stimuli (z = 2.10, *p* = 0.08). However, participants in the 40Hz-tACS_PRE group were significantly less accurate in perceiving scrambled stimuli than those in the 40Hz-tACS_ON group (mean = 0.695) (z = − 2.57, *p* = 0.027) (Fig. 6). Overall, since the critical sham versus 40Hz-tACS_ON scrambled did not reach significance, we can conclude that pareidolia for objects is not increased by 40 Hz tACS (or at least not as strong as that seen for faces). *Post-hoc* contrasts based on Stimuli showed that sham and 40Hz-tACS_ON groups did not differ between scrambled and inverted stimuli (*z* = 1.596, *p* = 0.24). In the 40Hz-tACS_PRE group however, accuracy for scrambled stimuli (mean = 0.618) was significantly lower than for the inverted (mean = 0.702) (*z* = − 4.946, *p* < 0.001), and upright ones (mean = 0.788), and between inverted and upright ones in all groups (all |*z|*s > 4, all *p*s < 0.001). This suggests an “Object inversion effect”.

*RTs* results indicate a main effect of Stimuli (F(2,8827.4) = 214.597, *p* < 0.001) and a Group*Stimuli interaction (F(4,8827.4) = 12.171, *p* < 0.001). *Post-hoc* contrasts based on Stimuli showed differences within all groups (all |*z|*s > 4, all *p*s < 0.001), with the exception of the contrasts between upright and inverted stimuli in the 40Hz-tACS_PRE (*z* = − 2.324, *p* = 0.052) and sham (*z* = − 2.275, *p* = 0.059) conditions. The difference between upright and inverted stimuli, with the formers recognized faster than the latter, was significant in the 40Hz-tACS_ON condition. Post-hoc contrasts based on Group did not reach significance (− 2.079 < *z*s < 0.945; all *p*s > 0.988).

### Toast test

**Figure 7:**
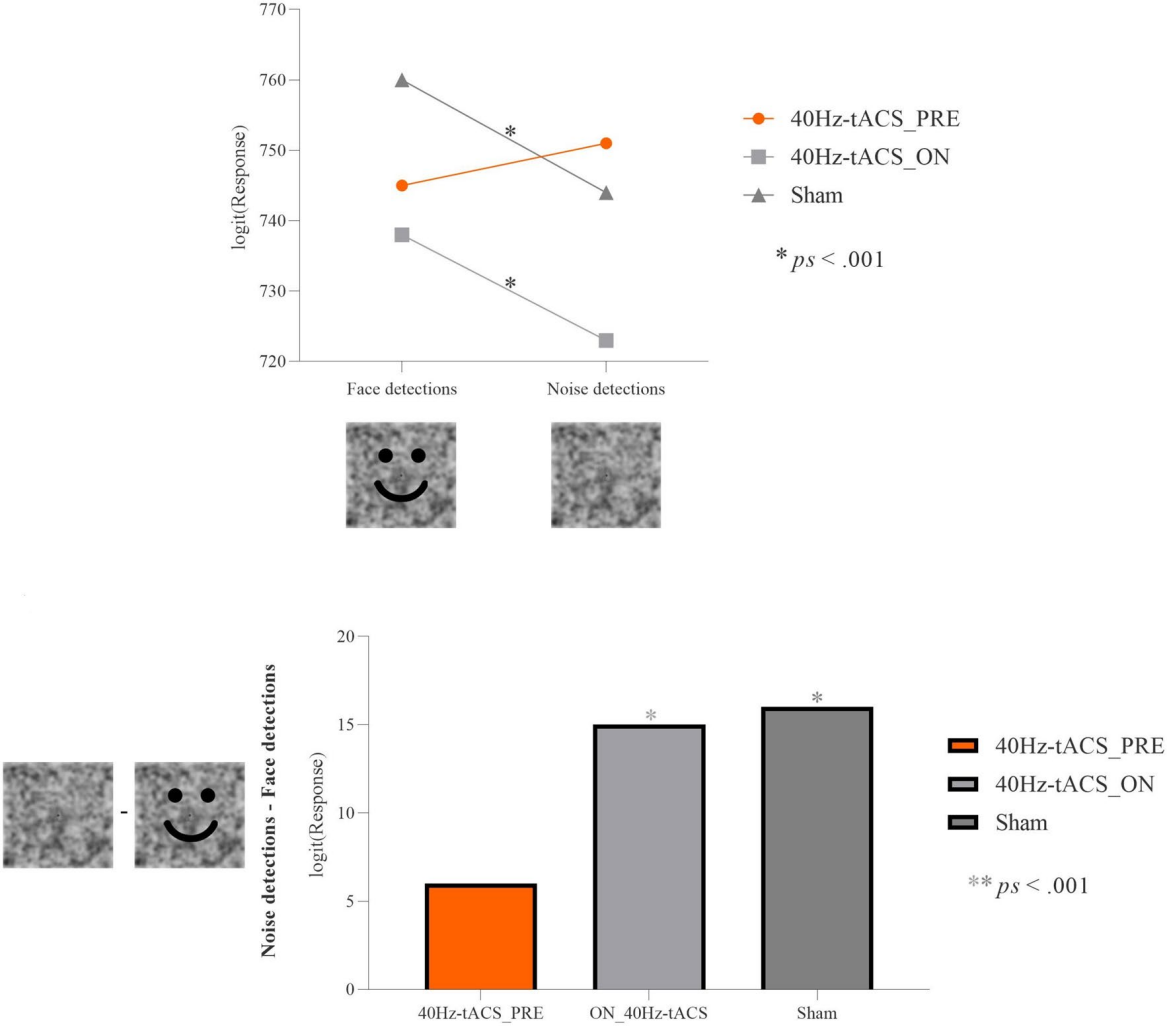
Group*Response interaction for the Toast test. Post-hoc contrasts based on Response. Upper part of the figure: RTs of pareidolia responses versus noise detections were not significantly different in the 40Hz-tACS_PRE group, while participants in the Sham and 40Hz-tACS_ON groups exhibited differential performances (i.e., significantly faster RTs for face detections vs. noise detections); bottom part of the figure: differences (in absolute values) between noise detections’ and face detections’ RTs among groups (40Hz-tACS_PRE =|6|; Sham =|16|; 40Hz-tACS_ON =|15|).

Overall, total *accuracy* rates (i.e., percentage of noise detections) were 39.7% (in line with results (34%) from the original work from^1^). Accuracy results showed no main effect of Group on Response (χ^2^(2) = 1.47, *p* = 0.48). RTs analyses showed a main effect of Response (F(1, 33,881) = 14.666, *p* < 0.001), with pareidolia responses (756 ms) having longer RTs than those of noise detections (743 ms). The Group*Response interaction on RTs was statistically significant (F(2, 33,881) = 6.5186, *p* = 0.001). Post-hoc contrasts based on Response showed that pareidolia responses took significantly longer than noise detections, both in the sham (pareidolia = 760 ms; noise = 744 ms; z = 3.830, *p* < 0.001) and in 40Hz-tACS_ON (pareidolia = 738 ms; noise = 723 ms; z = 3.461, *p* < 0.001) groups. By contrast, pareidolia responses in the 40Hz-tACS_PRE group (745 ms) were not different from noise perception (751 ms) (z = − 0.718, *p* = 0.473), showing that offline tACS induced faster pareidolia responses than sham and online stimulation, thus indicating a “pareidolia facilitation” (Fig. 7). Post-hoc contrasts based on the factor Group did not reach significance (all zs < 1; all ps > 0.09).

### Noise pareidolia test

**Figure 8:**
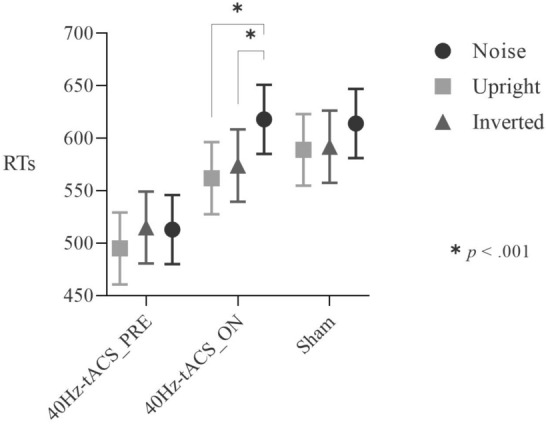
Group*Stimuli interaction for the Noise Pareidolia test. Post-hoc contrasts based on Stimuli. Participants in the 40Hz-tACS_ON group exhibited longer RTs for noise stimuli than for the upright and inverted ones. This suggests that online stimulation reduced participants’ speed in recognizing noise stimuli.

*Accuracy* results showed no effect of Group on Response (χ^2^(2) = 1.004, *p* = 0.605), and no Group*Stimuli interaction on Response (χ^2^(4) = 1.383, *p* = 0.847). RTs analyses showed a main effect of Stimuli (F(2,3919.9) = 17.256, *p* < 0.001) and a Group*Stimuli interaction (F(4,3920) = 3.2717, *p* = 0.011). *Post-hoc* contrasts based on Group did not reach significance (− 2.243 < *z*s < 2.159, *p* > 0.064). Contrasts based on Stimuli showed that only in the 40Hz-tACS_ON condition, RTs for noise stimuli (618 ms) were significantly longer than those for upright (589 ms) (*z* = 5.042, *p* < 0.001) and inverted stimuli (592 ms, *z* = 3.807, *p* < 0.001), indicating online tACS-induced slower rejections of pareidolia responses (See Fig. 8).

### Pareidolia task

*Accuracy* results from the Pareidolia task showed no significant effect of Group on Response (χ^2^(2) = 0.257, *p* = 0.879), nor a Group*Stimuli on Response (χ^2^(2) = 0.944, *p* = 0.623). Results from the RT analysis showed a significant main effect of Stimuli (F(1,5994.4) = 110.044, *p* < 0.001) as well as a significant Group*Stimuli interaction effect (F(2,5994.4) = 13.117, *p* < 0.001). *Post-hocs* based on Group were not significant (− 1.037 < *z*s < 0.839, all *p*s > 0.55), while contrasts based on Stimuli showed significantly larger RTs for landscapes compared to those for faces between each group (all |*z|*s > 2.4, all *p*s < 0.05), indicating that participants likely spent more time trying to identify faces in landscapes.

## Discussion

Face-pareidolia refers to the illusory phenomenon when typical subjects report face perceptions emerging from visual stimuli that do not contain any picture of a face. Albeit correlative evidence demonstrates that real face perception and face pareidolia share substantial neurophysiological features^25,26,70–73^, no causal evidence is available linking face-sensitive regions to face-pareidolia and, most importantly, linking specific neurophysiological activities to illusory face perception. Our results suggest the involvement of GBO in the “core” and “extended” face networks as neurophysiological mechanisms mediating illusory face perception.

### Anatomy and neurophysiology of the “real-face” perception network

Faces represent the stimuli we rely most on in social interactions since they convey crucial information about identity, emotions, approachability, age, race, and attractiveness^74^. With the exception of specific clinical conditions (e.g., prosopagnosia)^75–77^, humans are extremely proficient in face perception. Indeed, we can very quickly identify faces from natural scenes, even when their physical structure is partially occluded by objects or affected by light conditions^56,78,79^. This extraordinary ability to perceive, learn and recognise faces relies on a network of cortical and subcortical brain regions which can be grouped into the so-called “core” and “extended” systems^20,21,80^. The “core” system includes occipito-temporal areas such as the Occipital Face Area (OFA) in the lateral occipital cortex^81^, and the Fusiform Face Area (FFA) in the lateral fusiform gyrus^82^, which mediate bottom-up information transfer subserving face perception^18^. Albeit bilateral activation has been reported^19^, this system has strong right lateralization^17,83^. The “extended system”, including the PFC, consists of regions specialized for a wide range of high-order cognitive functions (e.g., attention, decision making)^19,20,30,84,85^. These areas provide top-down modulation into the visual cortex, so that retinal information is matched with memory templates and expectations during decision-making^30,86,87^. Specifically, the integration of visual information into the respective decision process (i.e., detecting the presence of a certain stimulus) and perceptual awareness involve activity in the PFC^32,88–90^.

The recent adoption of Noninvasive Brain Stimulation (NIBS) techniques with concurrent EEG/MEG can provide extensive *causal* evidence for the role of “core” and “extended” face networks in face perception^61,66,85,91–94^. It is now clear that, while people perceive faces, critical regions such as FFA and PFC are activated in less than 200 ms^38,40^, and predominantly exhibit oscillations at the gamma frequency^34,38,52,95^. Consistent evidence (even with beamforming source-reconstructed MEG—^96^) supports GBO involvement in holistic processing (i.e., faces perceived as a whole, and not as the sum of their features)^96–98^, in the construction of coherent representations from sensory inputs^99,100^, as well as in visual decision-making^101^.

### Neurophysiological features of “face-pareidolia”

What happens when people see faces in visual scenes/objects that do not contain any face representation (i.e., pareidolia)? Neuroimaging evidence demonstrates that pareidolic face illusions activate OFA/FFA and PFC, as also real faces do^1,26^, and that this “illusion” disappears after around 200 ms post-stimulus onset^27^. Since GBO are involved in the perception and construction of coherent representations from sensory inputs^99,100^, including those for complex visual stimuli such as faces^102,103^, gamma-tACS is potentially able to modulate illusory face perception^104^. Albeit no study has so far investigated GBO in face-pareidolia, it is possible that 40 Hz tACS entrainment of the face networks, by increasing feature-binding and perceptual integration^57,100^, might cause more “errors” (e.g., pareidolic illusions) and/or changes in RTs, where visual stimuli that do not contain any faces are more likely (and/or faster) perceived as faces. Indeed, the current study shows that tACS at 40 Hz modulates pareidolia.

Specifically, results of the present study from the Mooney test for faces show that participants in the 40Hz-tACS_PRE group were significantly less accurate in recognizing scrambled stimuli than those in the Sham and 40Hz-tACS_ON groups. In line with our expectations, given the higher rate of faces perceived in scrambled images, participants’ tendency to face pareidolia significantly increased (~ 10%) following 40 Hz stimulation. To control for the potential category-specificity of this effect, a control Mooney test for objects was conducted. Compared to the Mooney test for faces, results show that *offline* 40 Hz tACS did not lead to accuracy reduction in comparison with *sham*; thus, we can speculate that tACS pareidolic effects might have been stronger for faces. However, *offline* 40 Hz tACS caused more pareidolic object illusions than *online* 40 Hz tACS. This effect involving objects may reflect that (i) tACS at 40 Hz affects faces and objects perception differentially if online versus offline (as in^52^), (ii) GBO patterns characterize holistic integration irrespectively of the stimulus category^102,105^, and/or (iii) the adopted stimulation set-up affected brain activity in both face-specific and non-face-specific areas in the lateral occipital cortex.

Results from the Toast test (in which pure noise images *only* were shown), revealed that responses were longer for illusory face detections than for noise recognitions in the Sham and 40Hz-tACS_ON groups, suggesting participants’ hesitancy in responding that a face was present. By contrast, participants in the 40Hz-tACS_PRE group responded to non-face stimuli as fast as to those where a face was present. Thus, we showed that offline gamma-band stimulation over the fronto-occipito-temporal network affected the rate of pareidolic experiences versus noise detections differently than sham and online stimulation.

tACS effects on the Noise pareidolia test were observed *online* only. Indeed, participants in the 40Hz-tACS_ON group showed longer RT for noise as compared to upright and inverted stimuli. This means that *online* stimulation reduced speed in recognizing noise stimuli, which could represent a pareidolia-like effect, in which rejecting pareidolic responses while receiving 40 Hz tACS gets more difficult. No pareidolia-like effects of tACS emerged from the Pareidolia task, which might be the result of a ceiling effect. Indeed, young individuals seem to be highly accurate at face detection tasks for pareidolic faces, compared to elderlies^12^, which would imply that very demanding tasks are needed to investigate pareidolia in young healthy adults.

### Bottom-up or top-down modulations?

The experimental setup, as well as the spatial and temporal resolution of tACS can not provide definite information concerning whether the increased pareidolic effects reported are mediated mainly by bottom-up or top-down processing within the face network. Coupled modulation of *top-down* and *bottom-up* streams cannot be excluded, in light of the entangled forward and backward connections between frontal and posterior brain regions in determining visual perception and imagery (i.e., quick bidirectional interaction)^30,106^. However, 40 Hz tACS effects could have been primarily driven by enhanced *top-down* processes, from prefrontal areas to “core” face regions (i.e., stronger activation of PFC templates that modulate FFA/OFA activity). This is in line with evidence for PFC activity correlating with subjective awareness during visual recognition^32,87^, as well as with top-down connections determining the prediction of upcoming stimuli and the integration of their features^107^. Moreover, some studies showed dominant 40 Hz oscillations in decision-making and conflict monitoring networks (including the PFC) under difficult decision conditions (such as in our tasks), as compared to visual areas (exhibiting activity at higher frequencies)^108,109^. From a computational perspective, a prediction signal precedes the onset of stimuli (i.e., sensory input), and the prediction drives expectations, then predictions are tested on the basis of incoming sensory-driven information in a high-order manner, with attention increasing the influence of prediction signals on expectations^106,110^. Importantly, both attention and assessment of sensory predictions relie on GBO^111,112^. We can speculate that 40 Hz tACS modulated stimuli perception indirectly by boosting top-down processes, thus driving participants' proneness to perceptual illusions on the basis of expectations and decisional processes; this is in line with previous interpretations of behavioural findings for pareidolia^113^. Specifically, non-clinical illusory experiences, as well as clinical visual hallucinations, might stem from a highly biased balance between top-down and bottom-up perceptual processes toward top-down processes^113–116^.

### The relevance of *timing* in visual cognition

Effective modulation of visual perception mainly occurred in the *offline* group. What does a post-stimulation effect mean in terms of brain oscillations? tACS can exert its effects in terms of entrainment and/or plasticity^117,118^. It has been suggested that spike-timing dependent plasticity (STDP) involving N-methyl-D-aspartate receptor (NMDA-R) activity may be the mechanism underlying tACS aftereffects^119^. In line with previous evidence in visual cognition^52^, our results suggest an aftereffect of 40 Hz tACS. However, an online effect emerged from the Noise pareidolia test. This might be interpreted in terms of the capability of tACS to directly entrain neuronal endogenous oscillations (i.e., enhancing perceptual and cognitive processes according to the frequency provided)^120^. However, the hypothesis that our stimulation protocol enhanced gamma activity in the face network, thus modulating perceptual high-order processing, needs to be further tested in future research (see Paragraph 4.6 below).

### Clinical implications

Despite commonly experienced by the healthy population^113^, pareidolia is linked to the proneness for hallucinations in various clinical conditions, such as Schizophrenia (ScZ), Parkinson’s disease (PD), and Lewy-body dementia (LBD)^53,121–123^. Perception without the presence of an object is the classic definition of a hallucination^124^, while pareidolia misperceptions have been defined as a type of erroneous perception based on a real object in the external environment^125^. Since it is hard to differentiate short-lasting pareidolic illusions from psychotic-like hallucinations^126,127^, the former has been proposed as surrogate indicators of visual hallucinations, by reflecting a susceptibility to hallucinatory experiences^113,122^.

The relevance of the observed *offline* results stems from tACS potential to generate “stimulation-based” behavioural models of “psychotic-like” visual experiences. Indeed, our study shows that 40 Hz tACS modulates illusory perception towards an increase of healthy subjects’ pareidolia proneness, with effects outlasting stimulation. This provides further support for the role of gamma rhythms in perceptual integration^99,102,105^. ScZ represents a prime example of perceptual integration impairment, leading to higher rates of pareidolia than in healthy and other psychiatric populations^121,128^. Mooney stimuli (comparable to those used in our study) have been used to investigate GBO abnormalities in ScZ^40,41,129,130^. This disturbance suggests abnormal temporal integration of widely distributed brain networks (i.e., the *functional disconnection* model of ScZ), which stems from developmentally reduced synaptic connectivity and plasticity^131,132^. When perceiving Mooney stimuli, ScZ patients exhibit reduced accuracy and discrimination ability as a result of lowered bottom-up-driven sensory precision, compensated by excessive reliance on top-down information, which determines the patients’ tendency to assign meaning to irrelevant information^38,40^.

Non-invasive modulation of brain activity within areas devoted to visual perception and the construction of representations from sensory inputs might represent a valuable therapeutic avenue to reduce abnormal perceptual phenomena. Givent tACS potentiality to induce durable modifications suited for clinical applications (as shown in the current study)^133^, future research should pursue the objective of developing “pareidolia-reducing” protocols for patients exhibiting susceptibility to hallucinatory phenomena. Indeed, illusory experiences exert a detrimental impact on patients’ ability to engage in work, leisure, and self-care tasks^38,134^.

### Limitations and future directions

Potential limitations in the current study include the absence of a control stimulation montage and tACS frequency. Future research could extend our results by testing different brain areas (e.g., motor areas, to confirm the spatial specificity of our protocol’s effects), and/or by introducing other stimulation frequencies. This would allow testing for the frequency/localization specificity of the behavioural effects we have here shown. Despite counterbalancing the administration order of the tasks among groups might have prevented order effects, future studies should replicate our study with “pure” online versus offline groups. The absence of electrophysiological recordings represents a further limitation, precluding the concurrent investigation of tACS’ underpinning mechanisms. Indeed, future studies should implement a co-registration of EEG/MEG to elucidate the neurophysiological nature of the increased face pareidolia we demonstrated at the behavioural level, along with its spatio/temporal dynamics^135^.

### Conclusions

This is the first study investigating the neurophysiological mechanisms of face pareidolia. We demonstrate that 40 Hz tACS over the face network enhances face-like illusions (i.e., pareidolia) in healthy individuals. Our results have important applications for theories of face perception and pathophysiological processes involved in clinical conditions characterized by high rates of face pareidolia (e.g., first-episode ScZ, LBD, PD).

## Supplementary Information

Supplementary Information.

## Data Availability

The datasets generated and analysed during the current study are available in the ‘Raw Data’ repository (https://osf.io/fwtp7/?view_only=c4bc57e09d9946bc942265c3d96b29de).
