# Supplementary material for: Face pareidolia is enhanced by 40 Hz transcranial alternating current stimulation (tACS) of the face perception network
Source: Sci Rep. 2023 Feb 4;13:2035. doi: 10.1038/s41598-023-29124-8 (PMC9899232; doi:10.1038/s41598-023-29124-8)
Supplement: Supplementary file 1 — Supplementary Information. [file 41598_2023_29124_MOESM1_ESM.docx]

**Supplementary Materials**

**Face pareidolia is enhanced by 40 Hz transcranial Alternating Current Stimulation (tACS) of the face perception network**

Annalisa Palmisano^1^^, Giulio Chiarantoni^1^^, Francesco Bossi^2^, Alessio Conti^1^, Vitiana D’Elia^1^, Serena Tagliente^1^, Michael A. Nitsche^3,4^ & Davide Rivolta^1,5^

1. Department of Education, Psychology, and Communication, University of Bari Aldo Moro, Bari, Italy.
2. IMT School for Advanced Studies Lucca, Lucca, Italy.
3. Department of Psychology and Neurosciences, Leibniz Research Center for Working Environment and Human Factors (*IfADo*), Dortmund, Germany.
4. Department of Neurology, University Medical Hospital Bergmannsheil, Bochum.
5. School of Psychology, University of East London (UEL), London, UK

^ These authors contributed equally

**Corresponding author**:

**Annalisa Palmisano,** Ph.D. Candidate at University of Bari Aldo Moro, Bari, Italy **-** Department of Education, Psychology, and Communication. Phone: +39 3347560906; Email: annalisa.palmisano@uniba.it

**Running title:** 40Hz tACS enhances pareidolia

**Keywords**: tACS, pareidolia, visual illusions, faces, objects

1. **Descriptive Statistics**
   1. Descriptive statistics based on gender from the Toast Test

| Toast Test \| Response | | |
| --- | --- | --- |
| Mean | | |
|  | Female | Male |
| Sham | 0.604 | 0.566 |
| Online | 0.690 | 0.565 |
| Offline | 0.580 | 0.616 |
| Standard Deviation | | |
|  | Female | Male |
| Sham | 0.489 | 0.496 |
| Online | 0.463 | 0.496 |
| Offline | 0.494 | 0.487 |

| Toast Test \| Reaction Times | | |
| --- | --- | --- |
| Mean \| Female | | |
|  | Pareidolia | Noise |
| Sham | 778 | 759 |
| Online | 764 | 699 |
| Offline | 737 | 707 |
| Mean \| Male | | |
|  | Pareidolia | Noise |
| Sham | 743 | 707 |
| Online | 683 | 747 |
| Offline | 753 | 752 |
| Standard Deviation \| Female | | |
|  | Pareidolia | Noise |
| Sham | 255 | 270 |
| Online | 201 | 197 |
| Offline | 232 | 234 |
| Standard Deviation \| Male | | |
|  | Pareidolia | Noise |
| Sham | 295 | 318 |
| Online | 243 | 301 |
| Offline | 279 | 304 |

- 1. Descriptive statistics based on gender from the Mooney test for faces and the Mooney test for objects

| Mooney Faces \| Accuracy | | | |
| --- | --- | --- | --- |
| Mean \| Female | | | |
|  | Scrambled | Upright | Inverted |
| Sham | 0.659 | 0.918 | 0.741 |
| Offline | 0.484 | 0.904 | 0.779 |
| Online | 0.603 | 0.899 | 0.744 |
| Mean \| Male | | | |
|  | Scrambled | Upright | Inverted |
| Sham | 0.602 | 0.865 | 0.727 |
| Offline | 0.566 | 0.889 | 0.759 |
| Online | 0.627 | 0.880 | 0.746 |
| Standard Deviation \| Female | | | |
|  | Scrambled | Upright | Inverted |
| Sham | 0.474 | 0.274 | 0.439 |
| Offline | 0.500 | 0.295 | 0.415 |
| Online | 0.489 | 0.301 | 0.437 |
| Standard Deviation \| Male | | | |
|  | Scrambled | Upright | Inverted |
| Sham | 0.490 | 0.342 | 0.446 |
| Offline | 0.496 | 0.315 | 0.428 |
| Online | 0.484 | 0.326 | 0.436 |

| Mooney Faces \| Reaction Time | | | |
| --- | --- | --- | --- |
| Mean \| Female | | | |
|  | Scrambled | Upright | Inverted |
| Sham | 657 | 565 | 591 |
| Offline | 613 | 475 | 504 |
| Online | 658 | 498 | 537 |
| Mean \| Male | | | |
|  | Scrambled | Upright | Inverted |
| Sham | 641 | 536 | 568 |
| Offline | 670 | 544 | 558 |
| Online | 644 | 514 | 533 |
| Standard Deviation \| Female | | | |
|  | Scrambled | Upright | Inverted |
| Sham | 164 | 151 | 168 |
| Offline | 139 | 109 | 126 |
| Online | 141 | 112 | 149 |
| Standard Deviation \| Male | | | |
|  | Scrambled | Upright | Inverted |
| Sham | 162 | 168 | 212 |
| Offline | 196 | 179 | 195 |
| Online | 175 | 160 | 180 |

| Mooney Objects \| Accuracy | | | |
| --- | --- | --- | --- |
| Mean \| Female | | | |
|  | Scrambled | Upright | Inverted |
| Sham | 0.647 | 0.813 | 0.706 |
| Offline | 0.623 | 0.806 | 0.690 |
| Online | 0.681 | 0.815 | 0.724 |
| Mean \| Male | | | |
|  | Scrambled | Upright | Inverted |
| Sham | 0.711 | 0.711 | 0.604 |
| Offline | 0.613 | 0.772 | 0.713 |
| Online | 0.709 | 0.769 | 0.658 |
| Standard Deviation \| Female | | | |
|  | Scrambled | Upright | Inverted |
| Sham | 0.478 | 0.390 | 0.456 |
| Offline | 0.485 | 0.396 | 0.463 |
| Online | 0.466 | 0.389 | 0.448 |
| Standard Deviation \| Male | | | |
|  | Scrambled | Upright | Inverted |
| Sham | 0.454 | 0.454 | 0.489 |
| Offline | 0.487 | 0.420 | 0.453 |
| Online | 0.454 | 0.422 | 0.475 |

| Mooney Objects \| Reaction Time | | | |
| --- | --- | --- | --- |
| Mean \| Female | | | |
|  | Scrambled | Upright | Inverted |
| Sham | 704 | 645 | 656 |
| Offline | 644 | 565 | 590 |
| Online | 752 | 641 | 664 |
| Mean \| Male | | | |
|  | Scrambled | Upright | Inverted |
| Sham | 664 | 626 | 645 |
| Offline | 700 | 619 | 624 |
| Online | 717 | 642 | 668 |
| Standard Deviation \| Female | | | |
|  | Scrambled | Upright | Inverted |
| Sham | 202 | 182 | 184 |
| Offline | 152 | 128 | 149 |
| Online | 177 | 158 | 170 |
| Standard Deviation \| Male | | | |
|  | Scrambled | Upright | Inverted |
| Sham | 193 | 183 | 183 |
| Offline | 222 | 203 | 214 |
| Online | 217 | 199 | 216 |

- 1. Descriptive statistics based on gender from the Noise Pareidolia test

| Noise Test \| Accuracy | | | |
| --- | --- | --- | --- |
| Mean \| Female | | | |
|  | Noise | Upright | Inverted |
| Sham | 0.735 | 0.760 | 0.667 |
| Offline | 0.784 | 0.811 | 0.684 |
| Online | 0.689 | 0.748 | 0.733 |
| Mean \| Male | | | |
|  | Noise | Upright | Inverted |
| Sham | 0.713 | 0.827 | 0.752 |
| Offline | 0.649 | 0.747 | 0.755 |
| Online | 0.687 | 0.695 | 0.568 |
| Standard Deviation \| Female | | | |
|  | Noise | Upright | Inverted |
| Sham | 0.441 | 0.429 | 0.474 |
| Offline | 0.412 | 0.394 | 0.467 |
| Online | 0.463 | 0.437 | 0.445 |
| Standard Deviation \| Male | | | |
|  | Noise | Upright | Inverted |
| Sham | 0.453 | 0.380 | 0.434 |
| Offline | 0.477 | 0.437 | 0.432 |
| Online | 0.464 | 0.463 | 0.498 |

| Noise Test \| Reaction Time | | | |
| --- | --- | --- | --- |
| Mean \| Female | | | |
|  | Noise | Upright | Inverted |
| Sham | 600 | 625 | 636 |
| Offline | 534 | 553 | 611 |
| Online | 630 | 591 | 594 |
| Mean \| Male | | | |
|  | Noise | Upright | Inverted |
| Sham | 652 | 616 | 619 |
| Offline | 510 | 525 | 514 |
| Online | 619 | 598 | 612 |
| Standard Deviation \| Female | | | |
|  | Noise | Upright | Inverted |
| Sham | 183 | 121 | 137 |
| Offline | 159 | 101 | 142 |
| Online | 180 | 133 | 128 |
| Standard Deviation \| Male | | | |
|  | Noise | Upright | Inverted |
| Sham | 200 | 186 | 191 |
| Offline | 205 | 179 | 218 |
| Online | 235 | 171 | 215 |

- 1. Descriptive statistics based on gender from the Pareidolia task

| Pareidolia Task \| Accuracy | | |
| --- | --- | --- |
| Mean \| Female | | |
|  | Landscape | Face |
| Sham | 0.963 | 0.802 |
| Offline | 0.948 | 0.760 |
| Online | 0.952 | 0.778 |
| Mean \| Male | | |
|  | Landscape | Face |
| Sham | 0.918 | 0.720 |
| Offline | 0.926 | 0.710 |
| Online | 0.953 | 0.753 |
| Standard Deviation \| Female | | |
|  | Landscape | Face |
| Sham | 0.188 | 0.399 |
| Offline | 0.222 | 0.427 |
| Online | 0.214 | 0.416 |
| Standard Deviation \| Male | | |
|  | Landscape | Face |
| Sham | 0.274 | 0.450 |
| Offline | 0.262 | 0.454 |
| Online | 0.211 | 0.432 |

| Pareidolia Task \| Reaction Time | | |
| --- | --- | --- |
| Mean \| Female | | |
|  | Landscape | Face |
| Sham | 638 | 629 |
| Offline | 583 | 569 |
| Online | 640 | 595 |
| Mean \| Male | | |
|  | Landscape | Face |
| Sham | 608 | 595 |
| Offline | 629 | 620 |
| Online | 648 | 603 |
| Standard Deviation \| Female | | |
|  | Landscape | Face |
| Sham | 159 | 161 |
| Offline | 124 | 120 |
| Online | 171 | 144 |
| Standard Deviation \| Male | | |
|  | Landscape | Face |
| Sham | 173 | 171 |
| Offline | 233 | 185 |
| Online | 236 | 202 |

1. **Results for fixed effects and random effects from each task**
   1. **Toast Test: Generalized mixed effect model on Response**

**Random effects**

| Groups Name | Variance | Std. Dev. |
| --- | --- | --- |
| Participant (Intercept) | 1.42 | 1.192 |

Number of obs: 33941, groups: participants, 75

**Fixed effects**

| Effect | Deviance (Full model) | Deviance (Null model) | Chi-square | Degrees of Freedom | P-value |
| --- | --- | --- | --- | --- | --- |
| Group | 39372 | 39373 | 1.4654 | 2 | 0.481 |

Signif. codes: < 0.001 ‘***’ 0.001 ‘**’ 0.01 ‘*’ 0.05 ‘.’ 0.1 ‘ ’ 1

- 1. **Toast Test: Linear mixed effect model on RTs**

**Random effects**

| Groups Name | Variance | Std. Dev. |
| --- | --- | --- |
| Participant (Intercept) | 46086 | 214.7 |
| Residual | 27090 | 164.6 |

Number of obs: 33941, groups: participants, 75

**Fixed effects**

| Effect | Sum of Squares | Mean of Squares | Num DF | Den DF | F value | P-value |
| --- | --- | --- | --- | --- | --- | --- |
| Group | 4455 | 2228 | 2 | 72 | 0.0822 | 0.921 |
| Response | 397313 | 397313 | 1 | 33881 | 14.6663 | < 0.001 *** |
| Group * Response | 353177 | 176589 | 2 | 33881 | 6.5186 | 0.001 ** |

Signif. codes: < 0.001 ‘***’ 0.001 ‘**’ 0.01 ‘*’ 0.05 ‘.’ 0.1 ‘ ’ 1

- 1. **Mooney Test: Generalized mixed effect model on Response**

**Random effects**

| Groups Name | Variance | Std. Dev. |
| --- | --- | --- |
| Participant (Intercept) | 0.1581 | 0.3976 |

Number of obs: 26808, groups: participants, 75

**Fixed effects**

| Effect | Deviance (Full model) | Deviance (Null model) | Chi-square | Degrees of Freedom | P-value |
| --- | --- | --- | --- | --- | --- |
| Group | 31006 | 31008 | 2.4323 | 2 | 0.296 |
| Stimuli | 31006 | 32039 | 1033.1 | 2 | < 0.001 *** |
| Experiment | 31006 | 31008 | 2.0126 | 1 | 0.156 |
| Group * Stimuli | 30652 | 30723 | 70.429 | 4 | < 0.001 *** |
| Experiment * Group | 30652 | 30657 | 4.7294 | 2 | 0.094 . |
| Experiment * Stimuli | 30652 | 30927 | 274.54 | 2 | < 0.001 *** |
| Experiment * Group * Stimuli | 30651 | 30652 | 1.0083 | 4 | 0.909 |

Signif. codes: < 0.001 ‘***’ 0.001 ‘**’ 0.01 ‘*’ 0.05 ‘.’ 0.1 ‘ ’ 1

- 1. **Mooney Test: Linear mixed effect model on RTs**

**Random effects**

| Groups Name | Variance | Std. Dev. |
| --- | --- | --- |
| Participant (Intercept) | 16271 | 127.6 |
| Residual | 16263 | 127.5 |

Number of obs: 17968, groups: participants, 75

**Fixed effects**

| Effect | Sum of Squares | Mean of Squares | Num DF | Den DF | F value | P-value |
| --- | --- | --- | --- | --- | --- | --- |
| Experiment | 26330436 | 26330436 | 1 | 17878 | 1619.0201 | < 0.001 *** |
| Group | 21891 | 10946 | 2 | 72 | 0.6730 | 0.513 |
| Stimuli | 28178452 | 14089226 | 2 | 17880 | 866.3259 | < 0.001 *** |
| Experiment * Group | 2702603 | 1351302 | 2 | 17878 | 83.0896 | < 0.001 *** |
| Experiment * Stimuli | 1831833 | 915916 | 2 | 17879 | 56.3184 | < 0.001 *** |
| Group * Stimuli | 1960741 | 490185 | 4 | 17880 | 30.1408 | < 0.001 *** |
| Experiment * Group * Stimuli | 32110 | 8028 | 4 | 17879 | 0.4936 | 0.741 |

Signif. codes: < 0.001 ‘***’ 0.001 ‘**’ 0.01 ‘*’ 0.05 ‘.’ 0.1 ‘ ’ 1

- 1. **Mooney Faces: Generalized mixed effect model on Response**

**Random effects**

| Groups Name | Variance | Std. Dev. |
| --- | --- | --- |
| Participant (Intercept) | 0.176 | 0.4196 |

Number of obs: 13432, groups: participants, 75

**Fixed effects**

| Effect | Deviance (Full model) | Deviance (Null model) | Chi-square | Degrees of Freedom | P-value |
| --- | --- | --- | --- | --- | --- |
| Group | 14858 | 14861 | 3.1546 | 2 | 0.207 |
| Stimuli | 14858 | 16009 | 1151.1 | 2 | < 0.001 *** |
| Group * Stimuli | 14816 | 14858 | 41.986 | 4 | < 0.001 *** |

Signif. codes: < 0.001 ‘***’ 0.001 ‘**’ 0.01 ‘*’ 0.05 ‘.’ 0.1 ‘ ’ 1

- 1. **Mooney Faces: Linear mixed effect model on RTs**

**Random effects**

| Groups Name | Variance | Std. Dev. |
| --- | --- | --- |
| Participant (Intercept) | 14709 | 121.3 |
| Residual | 12939 | 113.8 |

Number of obs: 9062, groups: participants, 75

**Fixed effects**

| Effect | Sum of Squares | Mean of Squares | Num DF | Den DF | F value | P-value |
| --- | --- | --- | --- | --- | --- | --- |
| Group | 16494 | 8247 | 2 | 72.0 | 0.6373 | 0.532 |
| Stimuli | 21112912 | 10556456 | 2 | 8983.9 | 815.8494 | < 0.001 *** |
| Group * Stimuli | 1092689 | 273172 | 4 | 8983.9 | 21.1120 | < 0.001 *** |

Signif. codes: < 0.001 ‘***’ 0.001 ‘**’ 0.01 ‘*’ 0.05 ‘.’ 0.1 ‘ ’ 1

- 1. **Mooney Objects: Generalized mixed effect model on Response**

**Random effects**

| Groups Name | Variance | Std. Dev. |
| --- | --- | --- |
| Participant (Intercept) | 0.1836 | 0.4285 |

Number of obs: 13376, groups: participants, 75

**Fixed effects**

| Effect | Deviance (Full model) | Deviance (Null model) | Chi-square | Degrees of Freedom | P-value |
| --- | --- | --- | --- | --- | --- |
| Group | 15878 | 15880 | 2.0169 | 2 | 0.365 |
| Stimuli | 15878 | 16038 | 160.26 | 2 | < 0.001 *** |
| Group * Stimuli | 15848 | 15878 | 30.176 | 4 | < 0.001 *** |

Signif. codes: < 0.001 ‘***’ 0.001 ‘**’ 0.01 ‘*’ 0.05 ‘.’ 0.1 ‘ ’ 1

- 1. **Mooney Objects: Linear mixed effect model on RTs**

**Random effects**

| Groups Name | Variance | Std. Dev. |
| --- | --- | --- |
| Participant (Intercept) | 19712 | 140.4 |
| Residual | 18004 | 134.2 |

Number of obs: 8906, groups: participants, 75

**Fixed effects**

| Effect | Sum of Squares | Mean of Squares | Num DF | Den DF | F value | P-value |
| --- | --- | --- | --- | --- | --- | --- |
| Group | 42611 | 21306 | 2 | 72.0 | 1.1834 | 0.312 |
| Stimuli | 7726996 | 3863498 | 2 | 8827.4 | 214.5968 | < 0.001 *** |
| Group * Stimuli | 876504 | 219126 | 4 | 8827.4 | 12.1713 | < 0.001 *** |

Signif. codes: < 0.001 ‘***’ 0.001 ‘**’ 0.01 ‘*’ 0.05 ‘.’ 0.1 ‘ ’ 1

- 1. **Noise Test: Generalized mixed effect model on Response**

**Random effects**

| Groups Name | Variance | Std. Dev. |
| --- | --- | --- |
| Participant (Intercept) | 0.8034 | 0.8963 |

Number of obs: 5881, groups: participants, 75

**Fixed effects**

| Effect | Deviance (Full model) | Deviance (Null model) | Chi-square | Degrees of Freedom | P-value |
| --- | --- | --- | --- | --- | --- |
| Group | 6416.7 | 6417.7 | 1.0054 | 2 | 0.605 |
| Stimuli | 6416.7 | 6427.7 | 10.976 | 2 | 0.004 ** |
| Group * Stimuli | 6415.3 | 6416.7 | 1.3826 | 4 | 0.847 |

Signif. codes: < 0.001 ‘***’ 0.001 ‘**’ 0.01 ‘*’ 0.05 ‘.’ 0.1 ‘ ’ 1

- 1. **Noise Test: Linear mixed effect model on RTs**

**Random effects**

| Groups Name | Variance | Std. Dev. |
| --- | --- | --- |
| Participant (Intercept) | 26742 | 163.5 |
| Residual | 14605 | 120.9 |

Number of obs: 3999, groups: participants, 75

**Fixed effects**

| Effect | Sum of Squares | Mean of Squares | Num DF | Den DF | F value | P-value |
| --- | --- | --- | --- | --- | --- | --- |
| Group | 63941 | 31970 | 2 | 73.4 | 2.1890 | 0.11929 |
| Stimuli | 504053 | 252027 | 2 | 3919.9 | 17.2561 | < 0.001 *** |
| Group * Stimuli | 191132 | 47783 | 4 | 3920.0 | 3.2717 | 0.011 * |

Signif. codes: < 0.001 ‘***’ 0.001 ‘**’ 0.01 ‘*’ 0.05 ‘.’ 0.1 ‘ ’ 1

- 1. **Pareidolia Task: Generalized mixed effect model on Response**

**Random effects**

| Groups Name | Variance | Std. Dev. |
| --- | --- | --- |
| Participant (Intercept) | 0.4065 | 0.6376 |

Number of obs: 7481, groups: participants, 75

**Fixed effects**

| Effect | Deviance (Full model) | Deviance (Null model) | Chi-square | Degrees of Freedom | P-value |
| --- | --- | --- | --- | --- | --- |
| Group | 5571.2 | 5571.5 | 0.2957 | 2 | 0.862 |
| Stimuli | 5571.2 | 6161.6 | 590.34 | 1 | < 0.001 *** |
| Group * Stimuli | 5570.3 | 5571.2 | 0.9443 | 2 | 0.624 |

Signif. codes: < 0.001 ‘***’ 0.001 ‘**’ 0.01 ‘*’ 0.05 ‘.’ 0.1 ‘ ’ 1

- 1. **Pareidolia Task: Linear mixed effect model on RTs**

**Random effects**

| Groups Name | Variance | Std. Dev. |
| --- | --- | --- |
| Participant (Intercept) | 19173 | 138.5 |
| Residual | 14191 | 119.1 |

Number of obs: 6071, groups: participants, 75

**Fixed effects**

| Effect | Sum of Squares | Mean of Squares | Num DF | Den DF | F value | P-value |
| --- | --- | --- | --- | --- | --- | --- |
| Group | 9757 | 4878 | 2 | 71.9 | 0.3437 | 0.710 |
| Stimuli | 1561682 | 1561682 | 1 | 5994.4 | 110.0447 | < 0.001 *** |
| Group * Stimuli | 372316 | 186158 | 2 | 5994.4 | 13.1177 | < 0.001 *** |

Signif. codes: < 0.001 ‘***’ 0.001 ‘**’ 0.01 ‘*’ 0.05 ‘.’ 0.1 ‘ ’ 1
